# Dominance and parent-of-origin effects of coding and non-coding alleles at the acylCoA-diacylglycerol-acyltransferase (*DGAT1*) gene on milk production traits in German Holstein cows

**DOI:** 10.1186/1471-2156-8-62

**Published:** 2007-09-24

**Authors:** Christa Kuehn, Christian Edel, Rosemarie Weikard, Georg Thaller

**Affiliations:** 1 Research Unit Molecular Biology, Research Institute for the Biology of Farm Animals (FBN), 18196 Dummerstorf, Germany; 2 Institute for Animal Breeding and Husbandry, Christian-Albrechts-University Kiel (CAU), 24098 Kiel, Germany

## Abstract

**Background:**

Substantial gene substitution effects on milk production traits have formerly been reported for alleles at the K232A and the promoter VNTR loci in the bovine acylCoA-diacylglycerol-acyltransferase 1 (*DGAT1*) gene by using data sets including sires with accumulated phenotypic observations of daughters (breeding values, daughter yield deviations). However, these data sets prevented analyses with respect to dominance or parent-of-origin effects, although an increasing number of reports in the literature outlined the relevance of non-additive gene effects on quantitative traits.

**Results:**

Based on a data set comprising German Holstein cows with direct trait measurements, we first confirmed the previously reported association of *DGAT1 *promoter VNTR alleles with milk production traits. We detected a dominant mode of effects for the *DGAT1 *K232A and promoter VNTR alleles. Namely, the contrasts between the effects of heterozygous individuals at the *DGAT1 *loci differed significantly from the midpoint between the effects for the two homozygous genotypes for several milk production traits, thus indicating the presence of dominance. Furthermore, we identified differences in the magnitude of effects between paternally and maternally inherited *DGAT1 *promoter VNTR – K232A haplotypes indicating parent-of-origin effects on milk production traits.

**Conclusion:**

Non-additive effects like those identified at the bovine *DGAT1 *locus have to be accounted for in more specific QTL detection models as well as in marker assisted selection schemes. The *DGAT1 *alleles in cattle will be a useful model for further investigations on the biological background of non-additive effects in mammals due to the magnitude and consistency of their effects on milk production traits.

## Background

Several mapping studies revealed a QTL for milk production traits in the centromeric part of cattle chromosome 14 (BTA14) [1]. A positional and functional candidate gene approach detected that a non-conservative mutation (K232A) in the bovine acylCoA-diacylglycerol-acyltransferase 1 (*DGAT1*) gene is presumably the putative causal mutation for this QTL [2,3]. The postulated associations of the *DGAT1 K232A *alleles have been confirmed in several breeds and populations [4-6]. Further evidence for the hypothesis of the *DGAT1 K232A *locus representing the causal mutation for the QTL was achieved by functional in-vitro studies showing a higher efficiency in triglyceride synthesis of the allele *DGAT1 232K *compared to the allele *DGAT1 232A *[7]. However, consecutive studies suggested the presence of more than two alleles at the *DGAT1 *locus affecting milk production traits in cattle [8-10]. A VNTR polymorphism in the *DGAT1 *promoter containing a SP1 transcription factor binding site motif (CCCGCC) was targeted as functional background for these additional alleles due to the functional relevance of juxtaposed SP1 binding sites for the regulation of gene expression in eukaryotes [11,12]. The *DGAT1 *promoter VNTR alleles consisting of a variable number of putative SP1 transcription factor binding sites showed an association with daughter yield deviations for milk production traits in a data set comprising bulls with homozygous genotype *DGAT1 232A/232A *[9]. This result was particularly pronounced for milk fat percentage. The potential functional relevance of the *DGAT1 *promoter VNTR alleles is underlined by in-vitro studies providing evidence for SP1 binding to the CCCGCC motif in the *DGAT1 *promoter and for induction of gene expression by the *DGAT1 *promoter VNTR alleles [13].

There are several confirmed examples for non-additive QTL effects or QTL acting dependent of parental origin [14,15] in a variety of species, but the most prominent designs and approaches for QTL mapping do not allow detecting these phenomena. For example, if breeding values that are calculated on phenotypic observations in the individuals' offspring are used in the *per se *powerful granddaughter designs [16], it is impossible to model dominance or parent-of-origin effects [4]. Our studies on the effects of *DGAT1 *polymorphisms in a data set comprising direct trait measurement in cows, however, demonstrate that the *DGAT1 *K232A and the promoter VNTR alleles display a dominant-recessive pattern of effects on milk production traits. This pattern of effects was previously unnoticed in data sets using breeding values or daughter yield deviations. Additionally, we demonstrate differences in the effects of maternally and paternally inherited *DGAT1 *promoter VNTR – K232A haplotypes on milk production traits, which indicate a parent-of-origin effect at the *DGAT1 *locus in cattle.

## Results

### Allele and haplotype frequencies

Frequencies of the *DGAT1 *K232A and promoter VNTR alleles as well as of the *DGAT1 *promoter VNTR – K232A haplotypes based on the maternally inherited alleles/haplotypes are listed in Table 1. These represent the German Holstein field population, because no selection with respect to phenotypic traits had taken place while collecting the samples. The most frequent maternally inherited alleles are *DGAT1 232A *and the promoter VNTR allele *3*, respectively. All possible allele combinations were detected in the maternally inherited *DGAT1 *haplotypes with the maternally inherited promoter VNTR – K232A haplotype *3 – 232K *showing the highest frequency. Strong linkage disequilibrium was observed between both *DGAT1 *loci, because *DGAT1 232K *was almost exclusively detected in linkage phase with the promoter VNTR allele *3*. Due to their extremely low frequency, the *DGAT1 *haplotypes *1 – 232K*, *2 – 232K*, *4 – 232K*, and *5 – 232K *could not be included in the analysis of haplotype effects.

**Table 1:** Allele and haplotype frequencies for the *DGAT1 *promoter VNTR and K232A polymorphism

allele	*DGAT1 *promoter VNTR		allele	*DGAT1 *K232A		haplotype	*DGAT1 *haplotype^*b*^	
		
	pa^*a*^	ma^*a*^		pa^*a*^	ma^*a*^		pa^*a*^	ma^*a*^
*1*	0.06	0.02	*232K*	0.24	0.41	*1 – 232K*		0.001
*2*	0.13	0.15	*232A*	0.76	0.59	*2 – 232K*		0.030
*3*	0.34	0.55				*3 – 232K*	0.237	0.377
*4*	0.26	0.17				*4 – 232K*		0.007
*5*	0.21	0.11				*5 – 232K*		0.002
						*1 – 232A*	0.053	0.023
						*2 – 232A*	0.132	0.116
						*3 – 232A*	0.105	0.176
						*4 – 232A*	0.263	0.159
						*5 – 232A*	0.210	0.109

^*a *^allele frequencies were determined from the sires (pa) or from maternally inherited alleles/haplotypes of informative daughters (ma), ^*b *^*DGAT1 *promoter VNTR – K232A haplotype.

### *DGAT1 *promoter VNTR allele substitution effect

To enable a direct comparison of this study with results of a previous study in German Holstein sires [9], the gene substitution effects on milk production traits for the *DGAT1 *promoter VNTR allele *5 *compared to all other promoter VNTR alleles are listed in Table 2. Similar effects were found for the whole data set and for the subset of cows that were *232A/232A *homozygous. The *DGAT1 *promoter VNTR allele *5 *showed a significantly superior effect on milk fat content and milk protein content, while milk yield was decreased compared to all other promoter VNTR alleles. The differences for effects on milk fat yield and on milk protein yield were not significant.

**Table 2:** Effects of the *DGAT1 *promoter VNTR allele *5 *on milk production traits in German Holstein cows

	*DGAT1 232A/232A *only α^*ab *^(*S.E*.)	all individuals α^*ab *^(*S.E*.)
Milk yield [kg]	-88.0^ 0.068 ^*(48.0)*	-93.1^ 0.0074 ^*(34.7)*
Milk fat content [%]	0.0630^ 0.0002 ^*(0.0165)*	0.0752^ <0.0001 ^*(0.0124)*
Milk fat yield [kg]	1.79^ 0.319 ^*(1.79)*	2.44 ^0.075 ^*(1.37)*
Milk protein content [%]	0.0171 ^0.032 ^*(0.0079)*	0.0184^ 0.0016 ^*(0.0058)*
Milk protein yield [kg]	-1.56^ 0.271 ^*(1.41)*	-1.67^ 0.106 ^*(1.03)*

^*a *^average gene substitution effect of the *DGAT1 *promoter VNTR allele *5 *compared to all other alleles, where α is defined according to Falconer and Mackay [27]; ^*b *^p-values are given as indices.

### Effects of *DGAT1 *K232A and promoter VNTR genotypes

The *DGAT1 *K232A genotypes solitary showed highly significant effects on all milk production traits in a reduced model (See additional file 1: *DGAT1 *genotype effects on milk production traits). Similar results were observed with the full model including the *DGAT1 *K232A genotypes as well as the promoter VNTR genotypes. The only exception was for milk protein yield, where no significant effect for the *DGAT1 *K232A locus was obtained in the full model. In contrast to the lack of association between *DGAT1 *K232A genotypes and milk protein yield, the *DGAT1 *promoter VNTR genotype had significant effects on all milk production traits including milk protein yield in the full model.

The analysis of the contrasts between *DGAT1 *K232A genotype effects revealed that for all milk production traits the difference between *232A/232K *heterozygotes and the *232A/232A *homozygotes was larger than the difference between the *232A/232K *heterozygotes and the *232K/232K *homozygotes. This was most prominent for milk fat percentage in both models. Similarly, for milk yield and milk protein yield the difference between the *DGAT1 *promoter VNTR *4/4 *homozygotes and the */4 heterozygotes (* indicating any other promoter VNTR allele but allele *4*) was larger than the difference between the */4 heterozygotes and the */* homozygotes. The calculated dominance values showed significant dominance effects on milk yield, milk fat yield and milk protein yield (VNTR allele *4*), milk fat content (*DGAT1 232K*) and milk protein content (VNTR allele *5*) (Table 3). Heterozygotes for the VNTR allele *4 *had a lower milk yield and as a consequence a lower milk fat yield and a lower protein yield compared to the mean of the homozygotes with zero (*/*) or two copies (*4/4*) of the allele. Heterozygotes for the VNTR allele *5 *had a higher milk protein content and heterozygotes for the DGAT1 *232K *showed a significantly higher milk fat content and a tendency towards a higher milk protein content and to a reduced milk yield.

**Table 3:** Dominance values *(S.E.) *of *DGAT1 *K232A and promoter VNTR alleles for milk production traits in German Holstein cows

	*DGAT1 *allele		
	d_232*K*_^*a*^	d_*VNTR*4_^*a*^	d_*VNTR*5_^*a*^
Milk yield [kg]	-64.0^ 0.099 ^*(38.8)*	-153.8^ 0.001 ^*(47.7)*	-64.8^ 0.279 ^*(59.8)*
Milk fat content [%]	0.057^ <0.0001 ^*(0.014)*	0.020 ^0.263 ^*(0.017)*	0.032^ 0.140 ^*(0.021)*
Milk fat yield [kg]	2.18^ 0.157 ^*(1.54)*	-4.175^ 0.027 ^*(1.89)*	0.026^ 0.991 ^*(2.354)*
Milk protein content [%]	0.012^ 0.063 ^*(0.007)*	0.013^ 0.117 ^*(0.005)*	0.024 ^0.018 ^*(0.010)*
Milk protein yield [kg]	-1.17 ^0.313 ^*(1.16)*	-3.98^ 0.005 ^*(1.42)*	-0.21^ 0.906 ^*(1.78)*

Dominance values d is defined as difference between heterozygotes and the mean of the two homozygous genotypes. Regarding the promoter VNTR alleles, heterozygotes characterized by having one copy of the respective allele are compared to the mean of the groups of individuals with zero or two copies representing the alternative homozygous genotypes. ^*a *^p-values are given as indices.

### Effects of paternally and maternally inherited *DGAT1 *promoter VNTR – K232A haplotypes

The simultaneous test for a parent-of-origin effect over all haplotypes revealed a significant influence for the traits milk yield, milk fat content and a detectable, but not significant influence on milk protein yield (Table 4). More specifically, the *3 – 232K *haplotype resulted in significantly higher milk yield and milk protein yield and lower milk fat content when inherited paternally. In contrast to that, the *1 – 232A *haplotype resulted in significantly lower milk yield and higher milk fat content when passed by the sire to the offspring. For all other VNTR – K232A haplotypes, there is no influence of the parent-of-origin detectable at a significant level, but in the case of the *3 – 232A *haplotype, there were tendencies to a lower milk fat content and lower milk protein content for the paternally inherited haplotype. In a further analysis, we tested if those differences between paternally and maternally inherited *DGAT1 *haplotypes could also be detected when investigating the *DGAT1 *K232A alleles only. As indicated in Table 5, analysis of paternally and maternally inherited *DGAT1 *K232A alleles obtained results similar to those for the *DGAT1 *promoter VNTR – K232A haplotypes.

**Table 4:** Differences and their standard errors *(S.E.) *between paternally and maternally inherited *DGAT1 *promoter VNTR – K232A haplotypes regarding milk production traits in German Holstein cows

*DGAT1 *haplotype^*a*^	Milk yield [kg]	Milk fat content [%]	Milk fat yield [kg]	Milk protein content [%]	Milk protein yield [kg]
					
	pa – ma^*b*^	pa – ma^*b*^	pa – ma^*b*^	pa – ma^*b*^	pa – ma^*b*^
*3 – 232K*	324.44^ 0.001 ^*(98.2)*	-0.107^ 0.003 ^*(0.036)*	4.24^ 0.276 ^*(3.89)*	-0.023 ^0.149 ^*(0.017)*	8.93^ 0.002 ^*(2.93)*
*1 – 232A*	-467.13^ 0.019 ^*(198.47)*	0.20^ 0.006 ^*(0.07)*	-1.85 ^0.813 ^*(7.85)*	0.086^ 0.010 ^*(0.033)*	-8.16^ 0.169 ^*(5.92)*
*2 – 232A*	35.25 ^0.801 ^*(139.84)*	-0.050 ^0.330 ^*(0.050)*	-3.09^ 0.577 ^*(5.53)*	-0.029 ^0.223 ^*(0.024)*	-1.47^ 0.726 ^*(4.17)*
*3 – 232A*	139.48^ 0.318 ^*(139.56)*	-0.091 ^0.076 ^*(0.051)*	-1.94 ^0.726 ^*(5.52)*	-0.042 ^0.076 ^*(0.025)*	1.06^ 0.800 ^*(4.17)*
*4 – 232A*	-32.43 ^0.718 ^*(89.66)*	0.028 ^0.388 ^*(0.033)*	0.70^ 0.843 ^*(3.55)*	0.011^ 0.451 ^*(0.015)*	-0.20^ 0.939 ^*(2.68)*
*5 – 232A*	0.39 ^0.997 ^*(104.9)*	0.019 ^0.620 ^*(0.038)*	1.94 ^0.640 ^*(4.15)*	-0.003^ 0.854 ^*(0.018)*	-0.16^ 0.958 ^*(3.13)*
p	0.020	0.009	0.864	0.110	0.083

^*a *^*1, 2, 3, 4, 5*: alleles at the *DGAT1 *promoter VNTR; *K*: *DGAT1 232K*, *A*: *DGAT1 232A*; p: p-values for the F statistic of the simultaneous test of the parent-of-origin effect over all haplotypes. ^*b *^p-values for the contrast between single paternally (pa) and maternally (ma) inherited haplotypes are given as indices

**Table 5:** Differences and their standard errors (/S.E./) between paternally and maternally inherited /DGAT1/ K232A alleles regarding milk production traits in German Holstein cows

*DGAT1 *allele	Milk yield [kg]	Milk fat content [%]	Milk fat yield [kg]	Milk protein content [%]	Milk protein yield [kg]
					
	pa – ma^*a*^	pa – ma^*a*^	pa – ma^*a*^	pa – ma^*a*^	pa – ma^*a*^
*232K*	164.96^ 0.021 ^*(71.35)*	-0.045^ 0.087 ^*(0.026)*	3.13^ 0.269 ^*(2.83)*	-0.005^ 0.700 ^*(0.012)*	5.27 ^0.013 ^*(2.12)*

pa: paternally inherited allele; ma: maternally inherited allele;^*a *^p-values are given as indices

## Discussion

### Confirmation of *DGAT1 *K232A and promoter VNTR genotype effects

In our study, the *DGAT1 *promoter VNTR genotypes showed significant effects on all milk production traits in a model also including the *DGAT1 *K232A genotypes. Our results provide an independent confirmation of previous findings [8,9] demonstrating that the *DGAT1 *K232A mutation does not account for the entire QTL variation on milk production traits in the centromeric part of BTA14. These results are in contrast to Grisart *et al. *[7], who described that the *DGAT1 *K232A genotype did erase the effect of any other polymorphism at the *DGAT1 *locus. However, Grisart *et al. *did not consider the *DGAT1 *promoter VNTR. In our study, the K232A polymorphism did not significantly affect milk protein yield in a model that also included the promoter VNTR genotype. Compared to previous results obtained in an independent data set [9], the effects associated with alleles at the *DGAT1 *promoter VNTR showed an identical pattern and were very consistent in magnitude with allele *5 *being superior for milk fat and milk protein content. In an independent cattle breed, Sanders *et al. *[10] detected a regulation mechanism between the *DGAT1 K232A *mutation and the *DGAT1 *promoter VNTR for milk fat yield and content also supporting effects of those loci on milk production traits. Recently, Fürbass *et al. *[13] proved SP1 transcription factor binding and stimulation of gene transcription by the *DGAT1 *promoter VNTR motif. This observation and the confirmed trait association of *DGAT1 *promoter VNTR alleles presented in this study provide further evidence that the alleles at the *DGAT1 *promoter VNTR indeed may exert a direct effect on milk production traits. However, in spite of those data, it cannot be formally excluded that another, yet undetected gene outside the *DGAT1 *locus may be the molecular background for the additional QTL alleles in this genomic region.

### Dominance effects of *DGAT1 *genotypes

Previous publications on the effect of the *DGAT1 *locus focused on additive gene substitution effects for the QTL affecting milk production traits on BTA14 (e.g. [17]) as well as for the *DGAT1 *K232A mutation itself [3,4]. In contrast, our study, which based on a data set consisting of cows with direct phenotypic measurements, also allowed investigating non-additive effects and revealed significant dominance effects of the *DGAT1 *K232A alleles on milk fat content. This significant deviation from purely additively acting alleles has been detected in models for the *DGAT1 *K232A mutation only (data not shown) as well as in models combining the *DGAT1 *K232A and promoter VNTR genotype effects. Analogous to the non-additive effects of the alleles at the *DGAT1 *K232A locus, we obtained indication on dominance effects for the alleles *4 *and *5 *at the *DGAT1 *promoter VNTR locus. Interestingly, for the *232K *allele and the promoter VNTR allele *5*, which exhibit a similar pattern of effects on milk production traits, the pattern of dominance was also similar predominantly affecting milk fat and milk protein content. In contrast, recessive effects for the *DGAT1 *promoter VNTR allele *4 *were identified primarily on yield traits. Our data represent the first report on dominance effects on milk production traits for alleles at the *DGAT1 *locus.

Some studies [3,6,10,18] already investigated cow data sets facilitating tests for non-additive *DGAT1 *effects. Because Fisher and Spelman [18] focused on the detection of QTL by analysis of allele distortion within DNA pools of phenotypically extreme individuals, no estimation on the size and mode of allele effects is given. Studies in a New Zealand and an Israeli Holstein data set, respectively, did not find any indication on dominant effects [3,6]. However, Weller *et al. *[6] stated that the low number of homozygous cows with genotype *DGAT1 232K/232K *included in their study prevented a powerful analysis of *DGAT1 *K232A effects with respect to dominance. The dominant physiological effects of the *DGAT1 232K *variant on milk fat synthesis may be caused by the high efficiency of the 232K DGAT1 protein for triacylglycerol synthesis [7]. The high v_max _of the 232K DGAT1 molecules might result in the supply of substrates becoming the limiting factor for milk fat synthesis, thus preventing a further substantial difference in milk fat synthesis between individuals carrying one or two copies of the *DGAT1 232K *allele.

Dominant *DGAT1 *allele effects have an impact on allele substitutions effects evaluated by regression analysis on the number of *DGAT1 *K232A or promoter VNTR alleles. They can alter during selection processes in dependency of the magnitude of dominance and allele frequencies. However, allele effects obtained by regression in two independent populations (this study and [9]) showed a remarkable consistency in magnitude.

The discovery of dominant/recessive effects of *DGAT1 *alleles demonstrates that although indirect phenotypes like breeding values or daughter yield deviations may have an increased statistical power to detect QTL, the analyses of additional designs with direct phenotypes may be necessary for a precise estimation of the allele effects at causal mutations.

### Differences of effects between paternally and maternally inherited *DGAT1 *alleles/haplotypes

Our study revealed a difference between the paternally and maternally inherited *DGAT1 *promoter VNTR – K232A haplotypes regarding effects on milk yield, milk fat content and milk protein yield. The results for the differences between paternally and maternally inherited haplotypes are in line with results looking at alleles at the *DGAT1 *K232A locus only. These differences of effects between paternally and maternally inherited *DGAT1 *alleles/haplotypes have to be evaluated carefully to exclude artificial effects, e.g. due to the applied model including fixed effects of the sires, but not of the dams. This might result in a reduced variance of the paternally inherited alleles/haplotypes, because within the model the paternal *DGAT1 *haplotype effect might already be included in the sire effect. However, for the *DGAT1 3 – 232K *haplotype the associated effect on milk yield was larger when paternally inherited than when maternally inherited, while for *1 – 232A *the contrast was opposite. This result indicates that it is quite unlikely that a systematic bias due to reduced variance of paternally inherited alleles is the only source for the observed differences. We interpret these differences of haplotype contrasts for paternally and maternally inherited *DGAT1 *K232A – promoter VNTR haplotypes as parent-of-origin effects. If the *DGAT1 *locus indeed were a target for parent-of-origin effects, differences between paternally and maternally inherited alleles/haplotypes are expected for the *DGAT1 *promoter VNTR – K232 haplotype as well as for the *DGAT1 *K232A mutation alone as we found in our study.

It has to be noted that the *DGAT1 *gene is not located in a chromosomal region homologous to known imprinted genome areas in mice or human [19]. On the other hand, there seem to be differences in the imprinting pattern between human, mouse, and bovine genes [20,21]. However, as reviewed by [21], parent-of-origin effects are not necessarily the result of genomic imprinting. Further investigations to confirm the parent-of-origin effects detected in our study and to establish its molecular background within suitable resource populations are necessary. Interestingly, we observed parent-of-origin effects on lactation traits in a Charolais × German Holstein cattle F_2 _resource population [22], which will also be investigated in further *DGAT1 *studies.

## Conclusion

Our study indicates dominance and parent-of-origin effects at the *DGAT1 *locus in cattle and also provides further support for a functional role of the *DGAT1 *promoter VNTR additional to the *DGAT1 *K232A alleles on milk synthesis. The precise mechanisms behind the dominance and parent-of-origin effects require further targeted functional studies to be fully understood. Additionally, the *DGAT1 *allele effects deviating from the commonly applied hypothesis of additive QTL effects highlight the need towards efficient ways to include non-additive effects into QTL mapping models [23] and marker assisted selection programs [24]. The *DGAT1 *locus, which seems to be responsible for a substantial proportion of the genetic variance of milk production traits [3], demonstrates a prominent example for these needs. Particularly for traits, for which frequently indirect phenotypic measurements are used to achieve a high statistical power for detecting QTL effects, specific designs enabling the analysis of non-additive effects have to be set up to ascertain the genetic background of those traits. Due to the magnitude and consistency of associated effects, the investigation of the *DGAT1 *loci in cattle will be a useful model to study the biological background of dominance and parent-of-origin effects in mammals.

## Methods

### Animals

The data set of the study included a total of 1035 German Holstein cows sampled on ordinary dairy farms located in nine different regions of Germany. The cows descended from 19 different sires (41 – 101 daughters/sire, average family size 54.5 daughters/sire). The sires were pre-selected to represent a large variety of genotypes at the *DGAT1 *K232A and promoter VNTR polymorphisms. Nine of the sires were proven sires with high genetic merit for milk production traits with their daughters born after the sires finished their progeny test, whereas ten sires were young sires with their daughters originating from the progeny test itself. The cows themselves were unselected, except for their paternal ancestor.

### Markers

Within the *DGAT1 *gene the non-conservative polymorphism K232A in exon 8 giving rise to a lysine => alanine amino acid substitution as well as the non-coding VNTR in the promoter were included. The *DGAT1 K232A *mutation was genotyped according to [2]. PCR primers (*DGAT1*_UP _5'-GCACCATCCTCTTCCTCAAG-3'; *DGAT1*_DN _5'-GGAAGCGCTTTCGGATG-3') amplified a 411 bp fragment that was digested by the restriction enzyme *Cfr*I (MBI Fermentas, St. Leon-Rot, Germany). The resulting fragments (allele *232K*: one uncut fragment of 411 bp, allele *232A*: two fragments of 203 and 208 bp) were separated on a 2% agarose gel. A PCR fragment containing the VNTR polymorphism at position 1465 in the promoter of the bovine *DGAT1 *gene (Genbank no. AJ318490) was amplified by the following primers: *DGAT1*_proUP _5'-TCAGGATCCAGAGGTACCAG-3', *DGAT1*_proDN _5'-GGGGTCCAAGGTTGATACAG-3'. The PCR reaction was carried out in a 10 μl volume under the following conditions: 50 ng genomic DNA, 5 pmol of *DGAT1*_proUP_, 10 pmol of *DGAT1*_proDN_, 1.5 mM MgCl_2_, 0.2 mM of each dNTP, and 0.2 U Taq Polymerase (Qiagen, Hilden, Germany). A touchdown protocol was performed starting at 70°C with 2°C steps to the final annealing temperature of 60°C. One microliter of the reaction volume was run on an automated fragment analysis system (A.L.F., Amersham, Freiburg, Germany) under denaturing conditions. Allele nomenclature for the *DGAT1 *promoter VNTR was identical to allele classification in [9]. All genotypes were checked for plausibility and agreement with Mendelian inheritance.

*DGAT1 *promoter VNTR – K232A haplotypes of sires were derived from the genotypes of the respective group of daughters. Consecutively, the paternally inherited haplotype of each daughter was determined by assuming no recombination between *DGAT1 *promoter VNTR and K232A polymorphism due to the close genomic co-localization of both loci (8,968 bp distance, according to AJ318490; assuming a ratio of 1cM/1Mb, < 0.1 recombination is expected in the whole data set). The maternally inherited haplotype of a cow was deduced from subtracting the paternally inherited haplotype from the daughters' genotype. For daughters sharing their sires' genotype at the *DGAT1 *K232A as well as the promoter VNTR locus, it was not possible to assign paternally or maternally inherited haplotypes, respectively.

### Phenotypic data

For the 1035 cows included in the analysis, first lactation yield deviations (YD) [25] for milk yield, milk fat yield, and milk protein yield were available (Table 6). Yield deviations were the weighted average of a cows' yields adjusted for all non-genetic effects. YDs taken from the National genetic evaluation run for Holsteins in April 2005 [26] were not adjusted for the genetic merit of the dams to avoid elimination of the potential effects of maternally inherited *DGAT1 *alleles. Records were only included, if the cow had a minimum of eight milk recordings within the first lactation. Yield deviations for milk fat percentage and milk protein percentage were calculated according to [5].

**Table 6:** Phenotypic description of the data set

Yield deviation	n	minimum	maximum	mean	SD
Milk yield [kg]	1035	-881.6	3132.6	827.2	544.8
Fat content [%]	1035	-0.870	0.675	-0.099	0.230
Fat yield [kg]	1035	-36.29	113.76	26.03	21.49
Protein content [%]	1035	-0.305	0.294	-0.014	0.094
Protein yield [kg]	1035	-17.27	91.92	26.50	15.78

Minimum, maximum, mean, and standard deviation (SD) for first lactation yield deviations of German Holstein cows included in the study

### Statistical analysis

The allele and haplotype frequencies for the German Holstein population cannot be estimated from the genotypes of the cows, because the sires of the cows included in the study had been selected with respect to *DGAT1 *K232A and promoter VNTR genotypes to ensure a high variety of alleles and haplotypes in the analysis. Instead, the maternally inherited alleles and haplotypes of the cows were used to receive an overview of the *DGAT1 *allele distributions in the population.

In a previous study, daughter yield deviations (DYDs) of German Holstein bulls were analyzed, and the *DGAT1 *promoter VNTR allele *5 *displayed the most pronounced effect on milk production traits compared to the other VNTR alleles, particularly on milk fat percentage [9]. To enable the comparison of *DGAT1 *promoter VNTR effects estimated in that study with the effects within the German Holstein cow data set of the present study, the allele substitution effect of *DGAT1 *promoter VNTR allele *5 *compared to all other alleles was calculated using a regression analysis with general linear model procedures (SAS procedures GLM, SAS Institute, Cary, NC) applying the following model:

*y*_*ijk *_= μ + s_i _+ K232A_k _+ b*z_ij _+ e_ijk_

where *y*_*ijk *_is the YD of daughter j within sire i carrying the *DGAT1 *K232A genotype k, μ is the overall mean, s_i _is the fixed effect of sire i, K232A_k _is the fixed effect of the *DGAT1 *K232A genotype k; z_ij _is the number of alleles *5 *at *DGAT1 *promoter VNTR (0, 1 or 2) of daughter j within sire i, b is the regression coefficient representing the allele substitution effect of VNTR allele *5 *compared to all other alleles, and e_ijk _is the random residual effect. The respective analysis was performed including all individuals as well as considering a subset restricted only to the homozygous *DGAT1 232A/232A *cows.

While the previous study design consisted of *DGAT1 232A/232A *individuals only to evaluate *DGAT1 *promoter VNTR effects [9], the present analysis aims to estimate *DGAT1 *K232A and promoter VNTR effects jointly by including all *DGAT1 *K232A genotypes. General linear model procedures (SAS procedure GLM, SAS Institute, Cary, NC) were used to estimate the effects of both *DGAT1 *locus genotypes on milk yield, milk fat yield, milk protein yield, milk fat percentage, and milk protein percentage with the following model and a type III sum of squares test:

y_ijkl _= μ + s_i _+ K232A_k _+ VNTR_l _+ e_ijkl_

where y_ijkl _is the YD of daughter j within sire i carrying the *DGAT1 *genotypes k at K232A and l at the promoter VNTR, respectively, μ is the overall mean, s_i _is the fixed effect of sire i, K232A_k _is the fixed effect of the *DGAT1 *K232A genotype k, VNTR_l _is the fixed effect of the *DGAT1 *promoter VNTR genotype l, and e_ijkl _is the random residual effect. Only *DGAT1 *promoter VNTR genotypes represented by more than ten daughters in the data set were included in the analysis. For comparison to the outcomes of previous studies, a submodel of eq. 2 without the fixed effect of the *DGAT1 *promoter VNTR genotype was also used.

For both, the model and the submodel, the magnitude of the dominance was calculated by contrasting the estimated coefficients for heterozygotes to the average of the coefficient of homozygous genotypes. This was done for the *232K *allele and for the *DGAT1 *VNTR alleles *4 *and *5 *defining three genotype classes with zero, one or two copies of the respective allele in each case.

Finally, the effects of the maternally and paternally inherited *DGAT1 *promoter VNTR – K232A haplotypes on milk production traits were estimated separately applying the following model:

y_ijkl _= μ + s_i _+ DGAT1_pat_k _* DGAT1_mat_l _+ e_ijkl_

where y_ijkl _is the YD of daughter j of sire i with paternally inherited *DGAT1 *promoter VNTR – K232A haplotype k and maternally inherited *DGAT1 *promoter VNTR – K232A haplotype l, μ is the overall mean, s_i _is the fixed effect of sire i, DGAT1_pat_k _* DGAT1_mat_l _is the combined fixed effect of the paternally inherited *DGAT1 *promoter VNTR – K232A haplotype k and the maternally inherited *DGAT1 *promoter VNTR – K232A haplotype l, and e_ijkl _is the random residual effect. To test for a parent-of-origin effect, a set of linear hypotheses of the estimated effect coefficients were formulated that compare the estimates of the group that received a specific haplotype from the father to the estimates for the group that received it from the mother (equally weighing over the marginal occurrence of haplotype combinations similar to the so called least square means). Each linear hypothesis was tested separately (for each haplotype) and finally the whole set of hypotheses was tested simultaneously. This last test is interpreted as an overall test for the parent-of-origin effect. In this analysis, only observations with haplotypes occurring in both parent sexes were included.

## Competing interests

The author(s) declares that there are no competing interests.

## Authors' contributions

CK conceived, designed and coordinated the study, carried out the molecular genetic studies, performed a part of the statistical analyses and drafted the manuscript. RW participated in the molecular analysis and helped drafting the manuscript. CE performed the statistical analyses and GT participated in the statistical analysis and design of the study and helped drafting the manuscript. All authors read and approved the final manuscript.

## Supplementary Material

Additional file 1 ***DGAT1 *genotype effects on milk production traits**. Least square mean values *(S.E.) *for milk production traits for *DGAT1 *genotypes in a German Holstein cow data set from a combined model with *DGAT1 *K232A and VNTR genotype effects and from a reduced model including *DGAT1 *K232A genotype effects only. Click here for file
