# Supplementary material for: Dominance and parent-of-origin effects of coding and non-coding alleles at the acylCoA-diacylglycerol-acyltransferase (DGAT1) gene on milk production traits in German Holstein cows
Source: BMC Genet. 2007 Sep 24;8:62. doi: 10.1186/1471-2156-8-62 (PMC2129099; doi:10.1186/1471-2156-8-62)
Supplement: Additional file 1 — DGAT1 genotype effects on milk production traits. Least square mean values (S.E.) for milk production traits for DGAT1 genotypes in a German Holstein cow data set from a combined model with DGAT1 K232A and VNTR genotype effects and from a reduced model including DGAT1 K232A genotype effects only. [file 1471-2156-8-62-S1.pdf]

Table 3 - Least square mean values *(S.E.)* for milk production traits for *DGAT1* genotypes in a German Holstein cow data set

DGAT1 genotype[a]	Milk yield [kg]		Milk fat content [%]		Milk fat yield [kg]		Milk protein content [%]		Milk protein yield [kg]	
model[b]	I	II	I	II	I	II	I	II	I	II
K/K	605.1	690.0	0.088	0.1057	32.45	37.26	0.0286	0.038	22.66	26.22
	(49.0)	*(70.2)*	*(0.0178)*	*(0.0253)*	*(1.93)*	*(2.77)*	*(0.0082)*	*(0.0119)*	*(1.46)*	*(2.08)*
A/K	743.0	774.7	-0.0175	-0.0117	29.33	31.06	0.0011	0.0062	24.95	26.4
	(22.2)	*(35.6)*	*(0.008)*	*(0.0128)*	*(0.87)*	*(1.4)*	*(0.0037)*	*(0.006)*	*(0.66)*	*(1.05)*
A/A	997.3	987.5	-0.2411	-0.2438	21.09	20.5	-0.0482	-0.0501	29.42	28.92
	(24.5)	*(28.1)*	*(0.0089)*	*(0.0101)*	*(0.96)*	*(1.11)*	*(0.0041)*	*(0.0047)*	*(0.73)*	*(0.84)*
p	<0.0001	<0.0001	<0.0001	<0.0001	<0.0001	<0.0001	<0.0001	<0.0001	<0.0001	0.13
1/3		799.4		-0.1435		21.19		-0.0081		26.06
		(104.8)		*(0.0379)*		*(4.14)*		*(0.0178)*		*(3.12)*
1/4		812.0		-0.1234		23.24		0.0125		28.27
		(116.9)		*(0.0422)*		*(4.62)*		*(0.0198)*		*(3.48)*
2/2		809.3		0.0341		36.58		-0.0084		26.27
		(153.1)		*(0.0553)*		*(6.05)*		*(0.026)*		*(4.55)*
2/3		907.7		-0.0888		30.14		-0.0217		28.62
		(48.9)		*(0.0176)*		*(1.93)*		*(0.0083)*		*(1.45)*
2/4		770.2		-0.0585		26.94		0.0005		25.82
		(75.3)		*(0.0272)*		*(2.97)*		*(0.0127)*		*(2.24)*
2/5		691.3		0.0253		31.12		0.0329		25.85
		(74.1)		*(0.0268)*		*(2.93)*		*(0.0125)*		*(2.2)*
3/3		731.4		-0.0666		24.83		-0.0112		23.6
		(36.1)		*(0.013)*		*(1.42)*		*(0.0061)*		*(1.07)*
3/4		820.5		-0.0799		27.35		-0.0103		26.68
		(40.1)		*(0.0145)*		*(1.58)*		*(0.0068)*		*(1.19)*
3/5		712.0		0.009		30.08		0.0061		24.23
		(45.2)		*(0.0163)*		*(1.78)*		*(0.0076)*		*(1.34)*
4/4		1162.6		-0.126		36.48		-0.0238		36.83
		(86.8)		*(0.0313)*		*(3.43)*		*(0.0147)*		*(2.58)*
4/5		813.5		-0.0102		32.75		0.0158		28.58
		(66.5)		*(0.024)*		*(2.62)*		*(0.0112)*		*(1.97)*
5/5		778.5		0.0292		34.58		-0.0074		25.35
		(108.0)		*(0.039)*		*(4.27)*		*(0.0183)*		*(3.21)*
p		0.0003		<0.0001		0.022		0.0112		0.0033

[a] Allele coding: K: *DGAT1 232K*, A: *DGAT1 232A*; *1, 2, 3, 4, 5*: alleles at the *DGAT1* promoter VNTR; [b] model I: sub model see eq. 2 in Methods, model II: full model see eq. 2 in Methods
